# An Exposition of the Signs and Symptoms of Pregnancy, the Period of Human Gestation, and the Signs of Delivery

**Published:** 1838-01-01

**Authors:** 


### An Exposition of the Signs and Symptoms of Pregnancy, the Period of Human Gestation, and the Signs of Delivery. By *W. F. Montgomery*, A.M., M.D., M.R.I.A., Vice-President and Professor of Midwifery in the King and Queen's College of Physicians in Ireland. London, 1837. pp. 344.

The subject matter of the present volume forms the substance of a very elaborate article in the Cyclopædia of Practical Medicine. That article has been amplified by its author, into the form under which it makes a second appearance in public—so that, in fact—as much of this volume as relates to "the signs and symptoms of pregnancy," is a second and improved edition of the former.

The introductory chapter is an addition to that article, and contains much useful, although but little original matter.

We shall analyze its contents specially, and condense the information scattered through the remaining chapters, so as to give our readers a succinct account of a matter, which has been little understood—and has led to much disgraceful and humiliating contradiction in medical witnesses.

hope in this way to prepare our readers for the study of Dr. Montgomery's work itself, which will be the study of the subject at large.

Until of late years medical jurisprudence formed no integral part of the study of medicine. Individual facts were stated, individual conjectures hazarded, men were hanged and women tortured, according to the ignorance of professional witnesses, and the judgment of an unprofessional jury.

Beck in America, and Smith in England, have the merit of having led to the present improved course of instruction, which renders the study of forensic medicine an essential preliminary to a diploma, or a degree.

We have ourselves assisted in the progress of this improvement—and desire now to make the requirements of our Halls and Colleges somewhat more than a dead letter, by taking up the subject deliberately; proposing to review, from time to time, some of the topics of more pressing necessity, involved in it.

The first in natural order is that which refers to the commencement of a man's personal existence—with all the questions arising therefrom, and we esteem it a very opportune circumstance that Dr. Montgomery's work on the Signs and Symptoms of Pregnancy, is the first to come under our notice.

In doing so we shall follow the Doctor as closely as possible, and notice, 1st, the effects of pregnancy—2ndly, the practical considerations connected therewith, and 3rdly, the precautionary measures required to protect the pregnant female from any injury likely to accrue to her therefrom:—And afterwards take up the consideration of its signs and symptoms.

1. *Effects of Pregnancy.*—Immediately on conception, the uterine system takes on a new action, characterized by vigorous efforts in every part to provide for the wonderful process of gestation in all its stages. *The supply of blood to the uterus becomes increasingly greater*—an infiltration of serum takes place in its tissue, increasing the bulk of the organ—softening its texture—and separating its fibres. Lymph is effused over its internal surface, lining its cavity with the decidua, between which and the false membranes elsewhere resulting from inflammatory action, there is a close analogy. The secretion of serum goes on rapidly to form the liquor amnii.

*There is a proportionate increase of nervous energy*, owing to the increasing size of the nerves of the uterus—involving all the organs of the body in one common sympathy with its increasing sensibility and consequent irritability, through the medium of the great sympathetic nerve which supplies the interior of the uterus, and connects that organ with the kidneys, stomach, breasts and head, so giving rise to a fretful and feverish uneasiness, alternate heat and chilliness, sick stomach, broken slumbers, languor, lassitude, and drowsiness.

*Menstruation is suspended.* The breasts become swollen and sensitive, there is acceleration of pulse, and blood drawn during the early months of pregnancy exhibits the buffed coat of inflammation.

But one of the most striking phenomena is *the acquisition by the uterus of a principle of growth,* which enables it to accommodate itself to the growth of its inmate, to "grow with its growth, and with its strength, increase," until, in the words of our author :—

"It attains to dimensions of such magnitude, and undergoes changes in its component structures so remarkable, that, whether considered absolutely or relatively, they present to our observation a series of phenomena at once the most extraordinary and beautiful of any that claim our admiration in the arrangements of the animal economy." p. 3.

The dimensions of the uterus in its virgin state, and at the close of gestation stand in remarkable contrast, and this it was which appears to have forced from the enthusiasm of Swammerdam, the epithet miraculum naturæ, applied by him to the astonishing growth of this organ.

| | In the Virgin Uterus. | At the end of the ninth month of pregnancy. |
|---|---|---|
| The length is | $2\frac{1}{2}$ inches. | from 12 to 14 inches. |
| The breadth | $1\frac{3}{4}$ ,, | from 9 to 10 ,, |
| From back to front | 1 ,, | from 8 to 9 ,, |
| Cavity | $\frac{11}{14}$ or $\frac{3}{4}$ of an inch. | 408 ,, |
| Superficies | 16 inches. | 339 ,, |

Shewing upwards of 500 per cent. increase of capacity, and a solid increase of substance of from $4\frac{1}{3}$ to 51 cubic inches, or in the ratio almost of 12 to 1.

Situated as the uterus is, it follows of course, that the so wide expansion of that organ, lifting it out of the pelvis, and raising it into the cavity of the abdomen, must disturb its relations with the other viscera, and their's with each other.

*The bladder is* thereby *affected*, and frequent micturition is often a consequence ; sometimes, retention of urine ; the former from the cause already mentioned—a common source of nervous sensibility; the latter, from mechanical pressure. Incontinence of urine is another consequence occasioned by the weight of the uterus resting upon the fundus of the bladder and projecting it over the pubis.

*The circulation is interfered with* by the pressure of the uterus upon the trunks of the veins which return the blood from the lower extremities and parts within the pelvis, giving rise to anasarcous swellings of the feet and legs, and effusions within the cavity of the peritoneum only in part, however, from the mechanical pressure, the greater activity of the exhalants, accounting, in part, also, for such swellings and effusions.

At a late period of gestation, the full-grown viscus presses the liver and stomach upwards, and carrying the diaphragm before them, diminishes the capacity of the chest, impedes respiration, and induces dyspnœa—at the same time rendering digestion imperfect, and occasioning slight icterus by arresting the flow of bile into the duodenum.

The ascent of the uterus out of the pelvis, is necessarily in an oblique direction forwards—whence if inconveniences arise to the female, important advantages likewise arise—for, during the period of gestation, the uterus with its contents is kept from gravitating to the bottom of the pelvis, and of prolapsing through the soft floor of that cavity. The total interruption of the functions of the bladder and rectum is also prevented; and, in the words of Dr. Montgomery, "by this position of the uterus, its longer axis, or the line in which its expulsive effort is directed when in action, is brought into coincidence with the axis of the abdominal aperture of the pelvis; so that when labour commences, the child is presented for entrance into that cavity in the direction the best possible to facilitate its transmission." 9.

2. *Practical Considerations; and* 3, *Precautionary Measures* connected

*with Pregnancy.*—The disposition to plethora prevailing throughout the general system during gestation, leads to affections which call for an anti-phlogistic treatment. This tendency to redundance is increased instead of diminished in the greater number of pregnant women by the prejudice which prevails respecting the need of such for a double portion of food.

This error of judgment in the article of diet, leads inevitably to an injurious effect upon the alimentary canal, itself almost always impaired in activity during gestation. And neglect of the bowels, in addition to the increased activity of the vascular system, which is natural and necessary, and, the injurious practice of over-feeding, which is unnatural and uncalled for, combines to produce inordinate secretion of the liquor amnii, with its frequent consequence, relaxation of the uterus after delivery—hemorrhage—convulsions—inflammation—and mania. Food, plain and nutritious in quality—moderate in quantity and capable of ready assimilation; the daily relaxation of the intestines, by mild aperients if necessary, to overcome the torpor induced at this time; and the enforcement of constant and regular exercise, are the best prophylactics against such evils. The advantages of exercise, and habits of activity, are finely illustrated in the ruddy health and easy labours of the peasant's wife, affording a comparison with the case of the woman of fashion, by no means more favorable to the wit and wisdom of the latter than to her health and happiness.

The augmented sensibility, as well as increased vascularity of the pregnant female, heightens her excitability, and renders her more easily affected by external impressions. The imagination and the passions, always readily excited in woman, become morbidly alive to excitement of every kind when she becomes pregnant, and therefore render it unsafe to expose her to the sight or hearing of aught whereby they may be lightly wrought upon. Morgagni mentions a case where grief and fear, on account of her husband's sudden death, so affected his wife then with child, that the motion of the fœtus instantly became languid, after that ceased—and in eight days after it ceased to be felt, the widow miscarried. The melancholy history of the house of Eli, recorded by Samuel, terminates with an instance where horror slew the mother while it spared the child. And Dr. Montgomery adds two cases from his own experience illustrative of the injurious effects of theatrical exhibitions—and of that intellectual curry of the West—novel reading; in the former instance causing fright, profuse flooding, alarming exhaustion, and abortion; in the latter, producing insanity by presenting to the imagination a picture of the insane, in a book containing a description of one of the Maisons de Santé in France.

The nervous system being also extremely impressible in pregnant women, teaches the necessity of withdrawing them from scenes of suffering or distress, from cases of infectious disease, and from the sight of disgusting objects.

The doctrine of effects produced upon the child by the imagination of the mother, fell into disrepute from the "lying wonders" by which it was attempted to be maintained. The doctrine is nevertheless true, and may be reasoned away by the logician, or laughed out of date by those who affect to reject whatever is inexplicable, but is more or less received by every practitioner whose experience in midwifery has been at all enlarged. Dr. Montgomery argues that if a mental impression upon the mother can destroy the life, it may reasonably be deemed capable of modifying the organization of the fœtus. There is a fallacy in this argument, however, which hardly needs to be pointed out—it is one thing to arrest development and destroy life—it is a widely different thing to give the very form and pressure of an external impression to the living moving creature after development and during growth. Dr. M. adduces one remarkable case from his own experience, and one well-authenticated case like his is enough to establish the fact, that, through the impressibility of the mother, wrought upon by the revolting spectacle of another's deformity, the deformity of that other may be communicated to the babe in her womb.

But not physically only, does this extreme nervousness affect the pregnant woman; nor only metaphysically, but likewise morally, turning, in very truth, sweet to bitter and bitter to sweet; changing the amiable to morose, and converting those of cross and crabbed temper to another and more loveable state of mind. This nervous irritability in some cases deprives the sufferer of sleep and rest, though not in reality of the refreshment which flows therefrom; broken slumbers during pregnancy sufficing to recruit the woman's strength and spirits, as fully as unbroken repose at other times.

The mental depression of the pregnant female occasionally assumes a more serious character, partaking of the symptoms of melancholia while gestation lasts, and terminating after delivery in protracted and even confirmed madness. According to Esquirol, strong congenital predisposition to madness may arise from a fright sustained by the mother during pregnancy. The revolution in France was prolific of many such cases. Dr. Burrows considered those "phantasies, called longings," to be "decided perversions or aberrations of the judgment, though perhaps the simplest modifications of intellectual derangement." Some women are, by the same author, affirmed to be insane on every pregnancy or lying-in—and Dr. Montgomery gives a case of mania occurring in eight successive pregnancies. On the other hand, we have cases recorded, where the spirit of a sound mind appeared to have returned with the return, and disappeared at the close of every pregnancy.

The tendency to mental derangement is much increased in cases where moral evil is superadded to physical predisposition.

"In the opinion of Esquirol, the moral causes affecting pregnant women, are in relation to the physical as 4 to 1, and of 92 cases of puerperal mania reported by him, 20 (?) were in unmarried women. How deplorable then must be the condition of the mind in a woman who, led astray by the profligate from virtue's paths of pleasantness and peace, and then abandoned, is compelled to consider her pregnancy as a curse instead of a blessing, and has, in addition to the ordinary troubles of that state, to bear up against the agony of disappointed hopes, of affections misplaced and cruelly abused, to endure the present scorn of society, and the anticipation of a still increasing shame, for which she is to find no 'sweet oblivious antidote' of power to 'pluck from the memory a rooted sorrow,' or 'raze out the written troubles of the brain.' How often has such a state of mind been followed by convulsions, or, ending in insanity, has armed with the weapon of suicide the once gentle hand of her, who, to use the words of W. Hunter, 'might have been an affectionate and faithful wife, a virtuous and honoured mother, through a long and happy life; and probably that very reflection raised the last pang of despair which hurried her into eternity.' I have myself seen instances of such miserable results, and one of them very lately."—*Montgomery*, p. 22.

We have quoted the above eloquent and feeling passage at length for the benefit of our younger readers, in the hope of giving them as great an abhorrence of seduction as we ourselves cherish for the undoer of female chastity.

Puerperal mania may be apprehended, when the aggravated form of hysteria has prevailed during pregnancy.

But many of the remarkable phenomena noticed above are not the usual concomitants of gestation, most of them are rare occurrences; some, remarkably so; and all, exceptions to the general rule, and therefore calling for marked attention, as probable indications of a morbid state of the system about to be manifested, or actually existing, and otherwise obscure; requiring us, moreover, to be on the watch to counteract the influence of the predisposition in pregnant women to be injuriously affected even by ordinary causes, and much more so by those of an impressive kind.

Pregnancy, indeed, cannot be considered as a state of disease, but as a condition in strict consistency with sound health; for, if with a few it has deserved the name of a nine-months malady, the many have passed through it without any or with but little inconvenience; while mothers are observed to enjoy better health than unmarried and barren women. And, in numerous instances, it has warded off disease from the parties, as in epidemics—while in others, it has stayed the ravages of diseases actually present, as in phthisis.

Dr. Montgomery adverts to the interesting fact, which very early attracted our own notice, that—

"When a pregnant woman labours under a malady which is to end fatally before the completion of her gestation, it almost invariably happens that a short time, generally a day or two, before her death, the uterine action is established, and the child born." 26.

An occurrence so frequently observed by our author, that he regards it, and we agree with him, "as a pre-ordained arrangement to prevent the unborn child from participating in the decease of the mother."

The occasional "simulation of disease" accompanying pregnancy is another curious and interesting fact, upon which, however, we have not room to enter.

The legal enactments for the protection and comfort of pregnant women have not improved, either in justice or humanity, with the advance of the world's age, and the boasted advance of civilization. No wonder! The Malthusian doctrines having reduced the value of human life below par, and degraded the condition of pregnancy to the very borders of criminality. Our silly forefathers, and those most egregious fools the ancient Greeks and Romans, committed a woful mistake, from the terrible effects of which political economy is only now delivering the world. *They* thought, with an old-fashioned author of former times, one of another people, that "children and the fruit of the womb" are "like arrows in the hand of a giant," and "happy he that hath his quiver full of them." *We* are instructed, by sager heads and lips more eloquent, to look upon population as a curse, and—facilis descensus averni!—upon pregnancy as a crime. Why should we extend protection to the unborn babe, who had better never be born. fathers did so, but they did it in their ignorance!

Sed seria a jocis. Dr. Montgomery ends his chapter upon pregnancy the expression of a *fear* somewhat tantamount to our already statement of *a fact.*

"In conclusion," he says, (p. 27) "I *fear* that, if we take a review former times, a conviction will be forced upon us not very flattering fancied superiority above our ancestors in our watchful care of our when pregnant, or in the legal provisions enacted for their protection comfort, in both of which respects the laws and customs of the periods *seem* to have greatly excelled, both in justice and humanity, which even at this day prevail amongst us." Why? will the worthy allow us to inquire; why does he only fear, and not boldly affirm, on such point as this? A mind of such a stamp as his should be above fear, ought not to feign it when the cause of truth and humanity is at Indignant rebuke of the miserable legislation of our day in such and cases, affecting the life of born and unborn alike, would better become eloquent and masterly pen than the maudlin affectation of an unreal hension.

### II. Signs and Symptoms of Pregnancy.

A more important, a more delicate, or a more difficult duty devolves upon the medical practitioner than that of deciding upon cases of or of suspected pregnancy, where the parties may be suspected of feigning or concealing its existence, according to their presumed motives practising concealment or imposition.

The *difficulty* of attaining to a right judgment in such cases is both sical and moral; the inadequacy of the several physical signs taken to determine the question putting the examiner's skill and intelligence the test—and the conflict of a mind tossed between the sense of guilt the dread of exposure in cases of concealment, and absorbed in the engrossing passions of avarice, or of self-preservation in cases of feigning putting the credibility of the principal witness herself beyond all powers belief.

The *delicacy* by which such a subject is itself invested, is greatly ened by the concomitant circumstances of an investigation involving, case may happen, the rights of persons or the rights of property, and the one hand exposing the innocent to unjust imputation, while other the shame of public infamy may be undeservedly kept from the truly guilty.

The *importance* of a duty which affects alike the fortunes, lives, characters of individuals, is sufficiently obvious. But neither should difficulties of the inquiry deter us from entering upon it, nor the of the investigation impede us in progressing with it to a certain final termination, nor the importance of its results affect with professional cowardice when called upon by proper authority, to pronounce the ment to which we have attained. Yet do they render it imperative upon inquirer to make the necessary examination with all practicable, with possible care.

The signs of pregnancy are collegible from a variety of sources. Of the presence or absence of some of those signs, report is the only evidence; but report is too frequently here, as elsewhere, a common liar, never to be relied on without a corroborative testimony; and such testimony is only to be found in existing changes of which the senses can take cognizance.

Dr. Montgomery groups these signs into Presumptive, Probable, and Unequivocal. The *Presumptive* comprehends (A) the constitutional affections resulting from the new action taken on by the uterus, and (B) the whole train of sympathetic changes and deviations in other organs excited by the uterine irritation. A bare enumeration of these will at once shew their fallacy as tests of pregnancy—every one of them being separately, and many conjointly, present in diseased conditions of the great organ of gestation. They are:—A. Suppressed menstruation; increased vascular action; increased and increasing nervous irritability; alterations in the countenance. B. The greater size of the breasts, tingling pains therein, the formation of areolæ, and the secretion of milk; irritability of stomach, followed by vomiting; and a variable and capricious appetite. The *Probable*, embracing the altered condition of the uterus, effecting as it does correspondent alterations in the os and cervix uterus, enlargement and prominence of the abdomen, and a change in the umbilicus. The *Unequivocal*, including the contents of the uterus so enlarged, whether a fœtus, where pregnancy is concealed, or organized substances, not the product of conception, where it is suspected or simulated.

Let us examine these in detail, under their respective groups.

### I. Presumptive Signs of Pregnancy.

*Suppression of the Menses.*—Conception, in healthy women, whose menstrual discharges have been regular, is followed by a suppression of the menses at the next returning period. This is the rule—but a rule, like every other, liable to exceptions. Conception may take place before menstruation has been established at all. Morgagni, Frank, Capuron, Low, Foderé, and Sir E. Home have placed on record examples of this. The returns of the menstrual periods are sometimes very irregular and protracted. Cases also occur in which conception has followed the suppression of the menses. And this suppression may arise from other causes than conception, such as disease, cold, privations, and strong mental emotions. In some rare instances, conception takes place after long suppression of the menses from disease. Dr. Montgomery mentions a case where there had been no menstrual discharge for two years previous to conception. A woman may conceive while nursing without any previous return of the catamenia, and we have at present a case of a woman who has had several children, and is again pregnant—who has been always pregnant or a nurse since her marriage, and has never experienced a return of the catamenia.

From what has now been said, it is plain that the *absence* of the catamenia is no sign of pregnancy upon which dependance can be placed. Dr. Montgomery enquires whether their *presence* is any evidence that the woman is not pregnant? and in opposition to the authority of Denman, Hamilton, and Schmitt, decides in the negative, and decides, we think, erroneously—affirming at the same time that he has himself met with several instances of menstruation occurring *once* after conception, and citing in confirmation of his view—the authorities of Johnson, Desormeaux, Puzos, Stein, Gardien, Dewees and Gooch. The objection that the discharges in such cases are not the menstrual fluid he dismisses very summarily, with the assertion that "the discussion of that question does not concern us here." But where then does it concern us? All parties admit that in certain instances, discharges similar in colour, quantity, and period, proceed from the vagina after conception—and the only question is—are those discharges true menstrual fluid? We answer, no!—the source of menstruation is no longer open—those discharges proceed from another source—they differ in *quality* from the menses. Indeed, he afterwards very nearly admits as much when he confesses to agreeing:

"——— with Dr. Hamilton in believing, that *many reputed cases of this kind have obtained credence for want of a sufficiently careful examination,* by which it would have been discovered that there were such marked differences between the discharges taking place during pregnancy, and those to which the woman was naturally subject, in the intervals of their returns, in their duration, and in their quality, as would of themselves suggest the probable existence of some altered state of the system."—p. 48.

Suppression of the menses, without injury to the general health, taking place in a healthy woman whose catamenia were previously regular, may be admitted as presumptive proof of the existence of pregnancy, and vice versa.

*Nausea and Vomiting.* The stomach frequently becomes irritable upon conception—and this irritability is, doubtless, a salutary attendant upon pregnancy. It may occur, however, from many other causes than pregnancy—and may arise from disease—although a very little care will generally enable the inquirer to distinguish the two kinds. Dr. Montgomery omits mention of that remarkable characteristic of the vomiting in pregnancy, its commencement and greatest violence in the mornings, and gradual subsidence and total cessation towards the evening.

*Salivation.* The sympathetic irritation which affects the stomach, sometimes extends to the salivary glands—and salivation is the consequence—but as this is the exception to the rule, its presence or absence, scarcely affirms anything relative to pregnancy. We have a case at present, where the mother of five children hesitates to believe herself pregnant because she does not salivate, having done so in all former times of child-bearing.

*Swelling of the Breasts.* Every one is acquainted with that beautiful provision in nature for the supply of food suited to the wants and weakness of the new-born babe, in the swelling breasts of the mother teeming with milk for her young. But, like all the other presumptive signs, the swelling of the breasts is only presumptive proof at best of pregnancy—the unaltered state of the same organs no positive proof of the absence of that condition. Suppression of the menses, or their retention by an imperforate hymen will cause the former—weakly and delicate conditions may induce the latter. The natural fulness of the breasts can hardly be mistaken.

*The Areola.* The altered condition of the areola is a sign upon which Dr. Montgomery lays considerable stress, and with good reason—" as being one of the most certain external indications of pregnancy, arising from the operation of sympathy." Rœderer's description of this change is very accurate, and by our author considered the most so of any he has met with :—" menstruorum suppressionem, mammarum tumor insequitur; quocirca mammæ crescunt, replentur, dolent interdum, indurescunt: venæ earum cœruleo colore conspicuæ, redduntur, *crassescit papilla, inflata videtur, color ejusdem fit obscurior, simili colore distinguitur* discus ambiens qui in latitudinem majorem expanditur, parvisque eminentiis, quasi totidem papillulis, tegitur." To these several circumstances may be added another, viz : a soft, moist state of the integument, which seems to be raised and turgescent; a state caused by infiltration of serum into the subjacent cellular tissue.

The earliest period at which this change is observable is not perhaps very distinctly ascertained, but Dr. M. has recognized it fully at the end of the second month, " at which time the alteration in colour is by no means the circumstance most observable, but the puffy turgescence (though as yet slight) not alone of the nipple, but of the whole of the surrounding disc, and the development of the little glandular follicles are the objects to which we should principally direct our attention." p. 60.

During the course of the two following months, a circle of varying intensity in individuals of different complexions surrounds the nipple; differing in diameter from an inch to an inch and a half. In Negro women it becomes jet black with a purple shade—and in monkeys acquires turgescence and a bright vermillion colour. The surface of the areola immediately around the base of the nipple, is studded over with small glandular follicles, numbering from 12 to 20, and projecting from the $\frac{1}{16}$th to the $\frac{1}{8}$th of an inch. The integuments covering the part are turgescent, softer, and moister than the surrounding portion, and on both may be seen, especially in dark-eyed, and dark-haired women, " numerous round spots or small mottled patches of a whitish colour scattered over the outer part of the areola, and for about an inch or more all round, presenting an appearance as if the colour had been discharged by a shower of drops falling on the part."—p. 62. This appearance is visible in the fifth month. About the sixth month and afterwards, the breasts appear cracked with whitish, silvery lines, which, once formed, continue permanent—and, however diagnostic in first, will not avail to discover subsequent pregnancies.

Circumstances occur, however, to modify the certainty of any conclusions we may come to from this sign. Pregnancy may exist, and the areola be deficient in one of its distinctive marks—the colour. In cases of abortion, the areolar tubercles shrink, and lose their sero-lactescent moisture. On the other hand, women, after recent miscarriage, may retain the true characters of this change—as is the case also with nurses. In some young females the areola assumes the same shade of colour so frequently observed round the eyes of women. Smellie, William Hunter, Gooch, Campbell and Blundell attached the utmost importance to this test, some of them placing reliance on the colour, others on the turgescence of the integument. Dr. Hamilton of Edinburgh considering it " the principle distinctive mark between the part during pregnancy, and at other times in women who have had a family, and in whom the areolar disc retains its dark colour."

*Milk in the Breasts.* The secretion of milk in the male breast, under the stimulus of a child's mouth, attested by the Bishop of Cork, Baron Humboldt, and, more recently, by Captain Franklin, sufficiently exposes the fallacy of this as a distinguishing sign of pregnancy. Milk has also been secreted, owing to morbid extensions of the uterus exciting the mammary sympathy. Sexual excitement has also induced this secretion. Nor is it a sign which, if not otherwise fallacious, would avail us much as a guide, seeing that milk is generally secreted after delivery—and if before, only then when less uncertain signs are present. Nevertheless, taken in connexion with other proofs, it is valuable, and confirmatory of the existence of pregnancy, especially in those cases, where there has been no previous conception.

### II. PROBABLE SIGNS OF PREGNANCY.

*Enlargement of the Abdomen.* This is a necessary consequence of the growth of the uterus during gestation; but one which is not obvious for the first two months, during which the abdomen, where there is no inflation, becomes flattened—the enlargement which is frequently visible at that early period, arising from inflation of the bowels, and requiring to be carefully distinguished from the result of pregnancy. In the third month, however, the increasing size is perceptible and proceeds steadily in proportion to the enlargement of the uterus. Where there is not much fat, the outline of the gravid uterus may be felt and traced, rising in the abdomen with the advance of pregnancy—and when, from fat, or any other cause, it may be impracticable to feel the uterine tumor and define its circumference, the cause of the enlargement may be ascertained to be something which renders the abdomen more solid to the touch than natural—while there is no indication of disease.

The abdomen is sometimes distended by accumulations of fat in the omentum or integuments. It is also enlarged in cases of dropsy. On the other hand the increased size may be concealed by dress, and an appearance of increased size may be occasioned by the protuberance forward of the sacro-vertebral curve. "It is, perhaps, still more important to recollect, that although pregnancy should exist, if the child die, the development of the uterus will be arrested, and the enlargement of the abdomen will not continue to increase, but, on the contrary, will sometimes diminish, the dead fœtus being retained in utero for several months, and the patient, although really many months pregnant, may not exhibit any increase of size beyond what is natural to her; or being near the end of her nine months, may not be larger than she was at four or five."—p. 96.

Nor should we be unmindful of the occurrence of cases, where the flow of the menses being prevented by structural impediments, or the imperforate state of some of the parts, as the hymen, the fluid is deposited in the uterus, distending it and the vagina, enlarging the abdomen, and raising the uterus as high as the umbilicus. The existence of such cases calls for the utmost caution and care in making the examination before announcing the diagnosis.

*State of the Umbilicus.* The umbilicus is, as if drawn inwards and downwards during the two first months of pregnancy, in consequence of the descent of the uterus, but with its ascent, there is a corresponding rise of the umbilicus;—in the third month it is natural;—in the fourth not so hollow as before conception;—in the fifth and sixth almost level with the surface around; and in the sixth or seventh altogether so;—while towards the close of the process it projects considerably.

This is a very important sign, upon which some authors have placed great reliance—how far any solid tumor enlarging the abdomen may be productive of the same effect, is yet doubtful. Dr. Montgomery states, from experience, "that a morbid tumour in the abdomen, of a size and elevation as great as those of the uterus in the seventh month, may co-exist with a perfectly depressed umbilicus."—p. 98.

*Changes in the Uterus.* In the unimpregnated state of this viscus, the os uteri projects from a quarter to half an inch into the vagina: the projecting part feels firm; is of a conical form; with a transverse opening at its termination. When conception has taken place, it becomes altered in texture, and increased in size and weight. It is fuller, rounder, and softer to the touch—and the orifice feels as if it was circular.

The irritation of the uterus which precedes menstruation effects, however, a similar change in its form and texture, to the above—so also, only in a more marked degree, polypus, hydatids, an accumulation of fluid within its cavity: while, in women of relaxed habit, or who have borne children, the lips of the os uterus are always soft and tumid, and so far separated as to admit the point of the finger readily. But although such changes are no criterion of pregnancy—their absence is clearly indicative of the absence of that state.

The change in the cervix uteri is less equivocal—that part becoming more and more abbreviated every month after the fourth.

The size, situation, and consistence of the impregnated uterus, cannot be distinguished for the first three months from the same circumstances in the unimpregnated organ—and after that, the proofs of pregnancy accumulate from every direction. Indeed, the main difficulty consists in detecting pregnancy during the four first months—after that, the business is sufficiently simple.

Among the probable signs, also, we should perhaps include, *the blueness of the vagina*, considered by Dr. Kluge of Berlin, and M. Jacquemin of Paris, as a sure test of pregnancy. But Dr. Montgomery observes that, while in some of the cases examined by him, its existence was very obvious, in others it was so slight as to be scarcely if at all perceptible. According to Dr. K. the discoloration commences in the fourth week of pregnancy, goes on increasing till delivery, and ceases with the lochia. M. Jacquemin found it in those prostitutes who were pregnant, when he conducted the examination of the genitals in prostitutes according to the Paris Police Regulations. M. Duchatelet was present on the occasion, and 4500 prostitutes were examined. The fallacy of this test may be inferred from the source of it; viz. vascular congestion or determination of blood to the parts. In a vagina examined by Dr. M. during menstruation, the purple hue of the mucous membrane was distinctly perceptible. And the practice of examining the orifice and internal surface of the vagina to ascertain the fitness of some of the lower animals to receive the male, has led to the same discovery of determination of blood to those parts, discolouring them, or rather heightening the colour to a dark purple. In Mr. Cruickshank's "Experiments to discover the Ova of Rabbits," &c. is the following passage to that effect:—"May 30, 1778. I took a female rabbit, hot (as the feeders term it) that is, ready to be impregnated, and disposed to receive the male. This they find out not by exposing her to the male, but by turning up the tail and inverting part of the vagina: *its orifice and internal surface are then as black as ink, from the great derivation of blood to these parts.*" We conclude our notice of this sign with the remarks of Dr. Montgomery.—"Should subsequent observations prove that healthy pregnancy is invariably accompanied by such an appearance, becoming visible within the first or second month, the fact would certainly be one of the most important additions ever made to our means of making a correct diagnosis in cases of early pregnancy, and *the more especially as it would be* applicable to a period, at which we have no other satisfactory means of discovering the existence of that condition; and might, occasionally, under peculiar circumstances, be resorted to with propriety and advantage."—p. 128.

### III. UNEQUIVOCAL SIGNS OF PREGNANCY.

These are, the presence and motions of a fœtus in utero;—the expulsion of an embryo from the uterus;—and the state of the uterus after death.

*The Presence and Motions of the Fœtus in Utero.* These may be felt upon external examination, when the mother has no consciousness of any thing of the kind. It has happened to Dr. Montgomery, and it has happened to others, in numerous instances, and both before and after "quickening," or that "first sensation experienced by the mother of the life of the child within her womb." But not only may a mother be unconscious of the growing treasure which she carries about with her, she may, more unhappily be betrayed into the mistake of imagining that she is with child when she is only big with gas, or serum, or coagula. The case of Queen Mary is one of painful yet laughable interest: in the words of the historian—"The Queen's extreme desire of having issue had made her fondly give credit to any appearance of pregnancy; and when the Legate was introduced to her, she fancied that she felt the embryo stir in her womb. Her flatterers compared this motion of the infant to that of John the Baptist who leaped in his mother's belly at the salutation of the Virgin. Despatches were immediately sent to inform foreign courts of this event; orders were issued to give public thanks; great rejoicings were made; the family of the young prince was already settled; for the Catholics held themselves assured that the child was to be a male; and Bonner, Bishop of London, made public prayers be said, that Heaven would please to render him beautiful, vigorous, and witty. But the nation still remained somewhat incredulous; and men were persuaded that the Queen laboured under infirmities which rendered her incapable of having children. Her infant proved only the commencement of a dropsy; which disappointment, conjoined with other annoyances of a domestic nature, so irritated the Queen that she totally lost her temper, and was guilty of some disgraceful acts of unjustifiable severity."—*Hume's History of England, Chap.* xxxvi.

But, however "*unequivocal*" such a symptom may in reality be, when really existing, the semblance of it may be so like the reality as to deceive even experienced practitioners, some of whom have supposed they felt the motions of the fœtus where there was no pregnancy. Accumulated menstrual fluid in the uterus caused Dr. Dewees to err thus, and Dr. Ingleby was similarly misled in a case of abdominal tumor by "a distinct crawling movement perceptible within it." A deceptious skill on the part of the woman simulating the motions of the child, may also prove a source of error to the medical inquirer. Joanna Southcott appears to have possessed this power, and by means of it to have kept up the delusion which she practised until the cheat could no longer be matter of doubt. Dr. Blundell mentions another case of the kind.

The time at which this motion may be felt has been variously estimated. Dr. Hamilton says, "four calendar months after conception;" Rœderer, that 80 out of 100 women quickened at the fourth month, while, of the remainder, some did so a month earlier; some, a month later. Dr. Montgomery's experience is that the greatest number of instances occur between the twelfth and sixteenth week after conception, or, "adopting another mode of calculation, between the fourteenth and eighteenth week after the last menstruation." The earliest interval known by Dr. M. was eleven weeks and two days; by Dr. Hamilton, eleven weeks. Some women do not quicken until a later period. Such were those mentioned by Baudelocque, who passed the sixth and even seventh month. According to Johnson, there are cases where the end of reckoning is near before the motions are felt. But a still more remarkable fact is, the total absence of any thing like this sensible motion during the whole of gestation. Dr. Campbell of Edinburgh, Leveat, Baudelocque, Gardien, Gooch, and Dr. Montgomery, state cases of the kind.

*Substances expelled from the Uterus.*—These may be an ovum, a mole, hydatids, the membrane produced in dysmenorrhœa.

*Ova.*—The utmost care and skill may fail to detect the true character of the ovum which is expelled within the first month. Afterwards it becomes distinct enough to be recognized by almost any one "who will take sufficient time to examine" it.

When expelled whole, the uterine decidua covers it, and may be known by the external surface thereof being rough, and penetrated with numerous foramina for the passage of the blood; while the internal is smooth, slightly corrugated, and shews only few foramina, and those extremely minute. Dr. Montgomery notices, as he supposes for the first time, another remarkable feature of this product, and as there is a claim to originality thus set up, we deem it only due to the author to give publicity to the discovery in his own words:—

"Repeated examinations have shewn me that there are, on the external surface of the decidua vera, a great number of small cup-like elevations, having the appearance of little bags, the bottoms of which are attached to or embedded in its substance; they then expand or belly-out a little, and again grow smaller towards their outer or uterine end, which, in by far the greater number of them, is an open mouth when separated from the uterus; how it may be while they are adherent, I cannot at present say. Some of them which I have found more deeply imbedded in the decidua were completely closed sacs. Their form is circular, or very nearly so; they vary in diameter from $\frac{1}{12}$th to $\frac{1}{6}$th of an inch, and project about the $\frac{1}{12}$th of an inch from the surface of the decidua. Altogether they give one the idea of miniature representations of the suckers of the cuttle-fish. They are not confined to any one part of the surface of the decidua; but I think I have generally found them most numerous and distinct on those parts of it which were not connected with the capillary rudiments of the placenta, and at the period of gestation which precedes the formation of the latter as a distinct organ. *They are best seen about the second or third month, and are not to be found at the advanced periods of gestation.* This outer coat may be found only partially adhering to the ovum, or entirely torn away and separated from it during its expulsion; but in either case these characters mark the true uterine decidua, and are not found in the products of disease." 133-4.

"I confess I am not prepared," he adds in a note, "to offer any very decided opinion as to the precise nature or use of these decidual cotyledons (for to that name their form, as well as their situation, appears strictly to entitle them); but from having on more than one occasion observed within their cavity a milky or chylous fluid, I am disposed to consider them reservoirs for nutrient fluids separated from the maternal blood, to be thence absorbed for the support and development of the ovum. This view seems strengthened when we consider that, at the early periods of gestation, the ovum derives all its support by imbibition, through the connexion existing between the decidua and the villous processes covering the outer surface of the chorion." 134.

Internal to the uterine is the reflex decidua, with its smooth outer, and filamentous inner surfaces, and the arborescent villous bond of union between it and the chorion. These villi are positive proofs of the product being an ovum.

*Moles.*—The discrepancies of opinion among authors on the nature of these products—some declaring them to be independent of sexual intercourse, others insisting that they never exist except when such intercourse has taken place—involve in very considerable doubt the question of impregnation, when a mole is the substance expelled from the uterus.

*Uterine Hydatids,* from the contrariety of opinions, subject the medical jurist to similar doubts and difficulties as in the case of moles. Madame Boivin has done much, however, towards a satisfactory settlement of the question whether they depend upon conception; and her researches go to establish the affirmative.

*The Membrane of Dysmenorrhœa.*—The habitual occurrence of the pains which accompany the discharge of this membrane—the irrespondence of the breasts to the state of pregnancy—and the absence of the usual symptoms of that state, are sufficient to distinguish this membrane from the products of conception. And, if any doubt exist, the substance of the membrane is itself deficient in the characters already described of the true decidua.

We have not left ourselves space for an analysis of several chapters in this volume, which, although connected with its professed topic, are unnecessary to its complete discussion, and can only take up what remains of the signs of pregnancy; viz.

*State of the Uterus and Uterine Appendages after Death.*—When gravid, it must be enlarged; its enlargement should correspond with the period of supposed pregnancy; and there ought to be discovered, distinctly and unequivocally, an ovum, or some of its component structures.

It may, however, be found enlarged and *empty*, with several of the changes accompanying gestation. In such a case attention must be turned elsewhere for proofs.

The presence of a corpus luteum never occurs but as a sequence of impregnation. We state this positively, although in contradiction to many weighty authorities, by whom the converse of the proposition has been maintained. But then it behoves the examiner to know what a corpus luteum is, and what it is not; the fatal mistakes and erroneous opinions upon this subject all arising from ignorance of the true characters of that substance.

On examining the ovaries of a woman recently pregnant, the one whence the germ has escaped differs from its fellow; it is to the eye larger, rounder, more vascular; to the touch, fuller and softer; while the larger size is the result of a projecting tumor swelling out at one part from the natural outline, like the projection of the cornea from the globe of the eye. The increased vascularity is confined chiefly to the limits of this protuberance, the colour of which, unlike the rest of the organ, is "a deep or dull-brownish yellow, seen through a slightly reddish medium." On the surface of the prominent part, a distinct cicatrix, as of a rent imperfectly united, is observable; and round it, to a small extent, the peritoneal coat appears as if abraded or removed by slight ulceration. Here the ovulum will have escaped from the ovary. The form and size of the corpus luteum are generally oval, the longer axis measuring from $\frac{4}{8}$ths to $\frac{5}{8}$ths of an inch, the shorter from $\frac{3}{8}$ths to $\frac{4}{8}$ths, occupying from a quarter to one half of the whole area of the ovary, according to the period of gestation, the size being in an inverse ratio to the advance of pregnancy. Its structure is glandular and lobulated, with slight convolutions, somewhat resembling a section of the human kidney, or, on a diminished scale, the centrum ovale of the brain. Its name tells us its colour. Within the first four months of pregnancy, its centre exhibits a small cavity in a strong white cyst (the inner coat of the Graafian vesicle). Beyond that period the sides of this cyst close, obliterating the cavity, and leaving in its stead an irregular white line, of a stellated character. This is visible with the last remaining traces of the corpus luteum, and is its most diagnostic character.

When gestation ceases, the corpus luteum disappears; but this disappearance is gradual, yet always within the period required to complete gestation after a subsequent conception.

Having stated what the corpora lutea are, we come to shew what they are not, and this we shall do in the words of Dr. Montgomery. In the virgin corpora lutea, as certain protuberances have been unhappily called,

"1. There is no prominence or enlargement of the ovary over them.

2. The external cicatrix is almost always wanting.

3. There are often several of them found in both ovaries, especially in subjects who have died of tubercular disease, such as phthisis, in which case they appear to be merely depositions of tubercle, and are frequently without any discoverable connexion with the Graafian vesicles.

4. They present no trace whatever of vessels in their substance, of which they are in fact entirely destitute, and of course cannot be injected.

5. Their texture is sometimes so infirm, that it seems to be merely the remains of a coagulum, and, at others, appears fibro-cellular, like that of the internal structure of the ovary ; but never presents the soft, rich, lobulated, and regularly glandular appearance which Hunter meant to express when he described them as 'tender and friable, like glandular flesh.'

6. In form they are often triangular, or square, or of some figure bounded by straight lines.

7. They never present either the central cavity, or the radiated or stelliform white line, which results from its closure." 245.

With this quotation we conclude by recommending the work before us to the attentive study of our younger professional brethren, and to the diligent perusal of all others. It is a valuable collection of important facts, a judicious compendium of the knowledge accumulated by many minds ; and will prove a real help to the medical practitioner as well as to the medical witness and jurist.

We have left unnoticed the chapters " On the Period of Human Gestation," " On the Signs of Delivery," and " On the Spontaneous Amputation of the Fœtal Limbs in Utero," with which the volume is enriched. The second only has any thing to do with the subject of the present article, and the first will be taken up hereafter, when an opportunity may offer of discussing the question respecting the duration of human pregnancy.

When a second edition of Dr. Montgomery's work shall be called for, we recommend to him the use of the pruning knife, and a reconsideration of the order in which his chapters and their contents are arranged. All the facts of the book might be compressed into one-third of the space they now occupy, and all the opinions of himself and others which are worth preserving, might be stated in half the words used for that purpose in the present edition.

We throw out this hint from a sincere desire to promote the diffusion of as valuable a mass of professional information as it has fallen to our lot to review, and from a deliberate conviction that the medical press has become so prolific of *books*, that the time is not far distant when those only will be bought which are small. What is wanted in every branch of the profession are books of *facts* (true, not false facts), and of deductions. And we cannot but think he would be doing the study of medicine a great service who would reduce to "Aphorisms" all that is certain, and to " Queries" all that is probable, in all the many branches of that most comprehensive and laborious subject. " Facts " would be a very good title for a summary of every ascertained fact, winnowed, like wheat, from the chaff of crude hypotheses and unripe theories. And, if the unreal things which in times past have originated mistakes, and are perpetuating them to the present hour, were thrown into one lump of learned lumber under the head of " Fictions," the benefaction to medical science and to the medical student would be complete.

